# Fluid shear stress regulates placental growth factor expression via heme oxygenase 1 and iron

**DOI:** 10.1038/s41598-021-94559-w

**Published:** 2021-07-21

**Authors:** Nabil A. Rashdan, Bo Zhai, Pamela C. Lovern

**Affiliations:** 1 Department of Molecular and Cellular Physiology, Louisiana State University, Shreveport, LA USA; 2 Department of Pediatrics, University of Pittsburgh, Pittsburgh, PA USA; 3 Department of Physiological Sciences, Oklahoma State University, 264 McElroy Hall, Stillwater, OK 74078 USA

**Keywords:** Cell signalling, Growth factor signalling, Biomedical engineering

## Abstract

Increased fluid shear stress (FSS) is a key initiating stimulus for arteriogenesis, the outward remodeling of collateral arterioles in response to upstream occlusion. Placental growth factor (PLGF) is an important arteriogenic mediator. We previously showed that elevated FSS increases PLGF in a reactive oxygen species (ROS)-dependent fashion both in vitro and ex vivo. Heme oxygenase 1 (HO-1) is a cytoprotective enzyme that is upregulated by stress and has arteriogenic effects. In the current study, we used isolated murine mesentery arterioles and co-cultures of human coronary artery endothelial cells (EC) and smooth muscle cells (SMC) to test the hypothesis that HO-1 mediates the effects of FSS on PLGF. HO-1 mRNA was increased by conditions of increased flow and shear stress in both co-cultures and vessels. Both inhibition of HO-1 with zinc protoporphyrin and HO-1 knockdown abolished the effect of FSS on PLGF. Conversely, induction of HO-1 activity increased PLGF. To determine which HO-1 product upregulates PLGF, co-cultures were treated with a CO donor (CORM-A1), biliverdin, ferric ammonium citrate (FAC), or iron-nitrilotriacetic acid (iron-NTA). Of these FAC and iron-NTA induced an increase PLGF expression. This study demonstrates that FSS acts through iron to induce pro-arteriogenic PLGF, suggesting iron supplementation as a novel potential treatment for revascularization.

## Introduction

Coronary artery disease (CAD) is a major cause of death worldwide^1,2^. A primary predictor of survival for CAD patients is the number of preexisting collateral vessels (arterial–arterial anastomoses) and the degree to which they have remodeled outward to increase their flow capacity^3,4^. This outward remodeling occurs in response to increased flow through the vessels, which is generated by an increase in the pressure gradient across the vessels due to decreased downstream pressure in the occluded branch. This remodeling process is termed arteriogenesis. Pharmacological stimulation of arteriogenesis has been a long sought after goal, because of the potential of arteriogenesis to reduce mortality and morbidity in CAD. However, early attempts to induce arteriogenesis via administration of single exogenous growth factors were marred with failure^5^, and it is clear that a deeper understanding of the myriad of signaling events contributing to arteriogenesis is necessary to develop safe and effective pro-arteriogenic treatments.

Placental growth factor (PLGF) is a member of the vascular endothelial growth factor (VEGF) family. PLGF is a potent arteriogenic agent, even more so than VEGF-A^6,7^. PLGF exclusively binds fms-like tyrosine kinase-1 (VEGFR-1) and elicits distinct downstream signaling events than those induced by VEGF-A binding of VEGFR-1^8^. PLGF exacts its arteriogenic effect by recruitment of monocytes (which only express VEGFR-1) to the vascular wall^6,9,10^. PLGF knockout mice exhibit a blunted arteriogenic response to hindlimb ischemia^6^, whereas PLGF protein levels in coronary artery plasma are positively correlated with improved patient outcome following myocardial infarction^11^.

Heme oxygenase (HO) catabolizes heme into equimolar quantities of CO, divalent iron, and biliverdin. There are two isoforms of HO; an inducible isoform (HO-1) and a non-inducible, constitutively expressed isoform (HO-2). HO-2 is constitutively expressed in the testes^12^ and the brain^13^. HO-1, on the other hand, is strongly induced in response to cellular stresses such as increased reactive oxygen species^14,15^ (ROS) and radiation^16,17^. There are only two reported cases of HO-1 deficiency in humans. These patients exhibited severe growth retardation and endothelial dysfunction, along with abnormal hemostasis and an increased susceptibility to oxidative stress^18–20^. HO-1 knockout mice present with similar growth abnormalities and appear to be in a chronic inflammatory state^21^. Arteriogenesis is diminished with advanced age^22^ and this effect may be related to abnormal HO-1 expression and/or signaling, since induction of HO-1 in aged rats with blunted arteriogenic potential restores outward vascular remodeling to levels comparable with young rats^23^.

We previously reported that exposure to arteriogenic FSS increases PLGF protein and mRNA both in vitro in a co-culture model of the vessel wall and ex vivo in isolated mouse mesenteric arterioles. We also reported that this increase is dependent on FSS-induced production of hydrogen peroxide by endothelial NADPH oxidase 4 (Nox4)^24^. It is established that FSS also increases activation and expression of HO-1 in endothelial cells^25–27^, and that these effects are dependent on ROS produced by Nox isoforms^25,27^. Similarly, HO-1 is upregulated in hindlimb skeletal muscle following femoral artery ligation^28,29^ in a Nox-dependent manner^29^. A possible link between HO-1 and PLGF is suggested by the observation that PLGF and HO-1 expression are both significantly increased in hindlimb skeletal muscle early after femoral artery ligation^30^. Furthermore, HO-1 haploinsufficiency in mice causes a decrease in PLGF expression and reduces the extent of revascularization following induction of hindlimb ischemia^31^. Lastly, HO-1 knockout increases the expression of antiarteriogenic soluble VEGFR-1^32^. Therefore, we hypothesized that the effects of FSS on PLGF are mediated by HO-1. We tested this hypothesis in an endothelial cell/smooth muscle cell co-culture model and in isolated mouse mesenteric arterioles in order to characterize the role of HO-1 in FSS-mediated regulation of PLGF.

## Results

**Figure 1:**
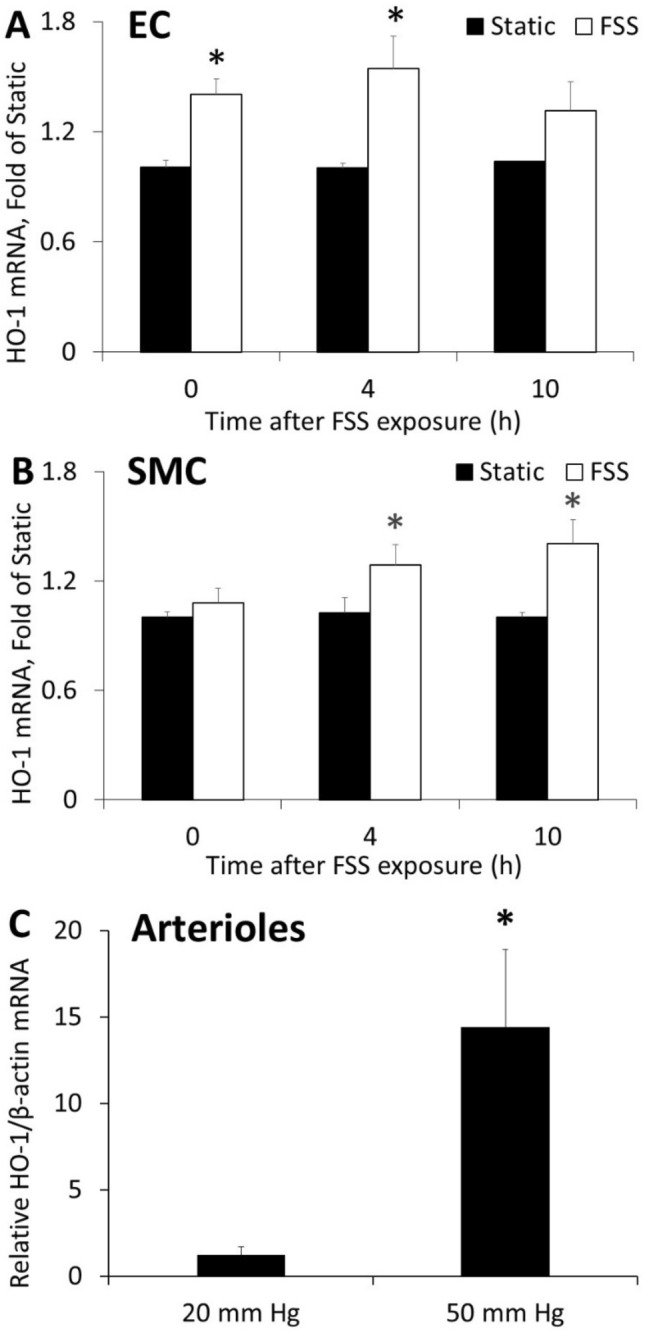
FSS increases HO-1 expression. Endothelial/smooth muscle co-cultures were exposed to FSS for 2 h and samples were collected immediately after exposure (0 h), 4 h later, and 10 h later. (**A**) In endothelial cells, FSS increased HO-1 mRNA compared to static control at 0 h and at 4 h but not at 10 h (n = 5, p < 0.05). (**B**) In smooth muscle cells co-cultured with endothelial cells exposed to FSS, HO-1 mRNA was increased at 4 h and 10 h (n = 5, p < 0.05). (**C**) In mesenteric arterioles, perfusion at a pressure gradient of 50 mmHg increased HO-1 mRNA compared to perfusion at 20 mmHg (n = 5, p < 0.05).

First, we determined the relative mRNA levels of HO-1 in our models following experimental treatment. In endothelial cells (Fig. 1A), shear stress significantly increased HO-1 mRNA immediately after exposure (1.40 ± 0.08-fold of static control), and this increase remained evident 4 h after exposure (1.54 ± 0.18-fold of static). By 10 h after exposure, HO-1 mRNA was not significantly different from control. In smooth muscle cells (Fig. 1B), HO-1 mRNA was significantly increased 4 h after exposure to shear stress (1.29 ± 0.11 fold of static) and remained elevated 10 h post exposure (1.41 ± 0.13-fold of static). Mouse mesenteric arterioles (Fig. 1C) exposed to elevated flow (50 mmHg pressure gradient) expressed significantly higher levels of HO-1 mRNA compared to vessels subjected to normal flow (20 mmHg pressure gradient) (14.43 ± 4.48-fold of 20 mmHg).

**Figure 2:**
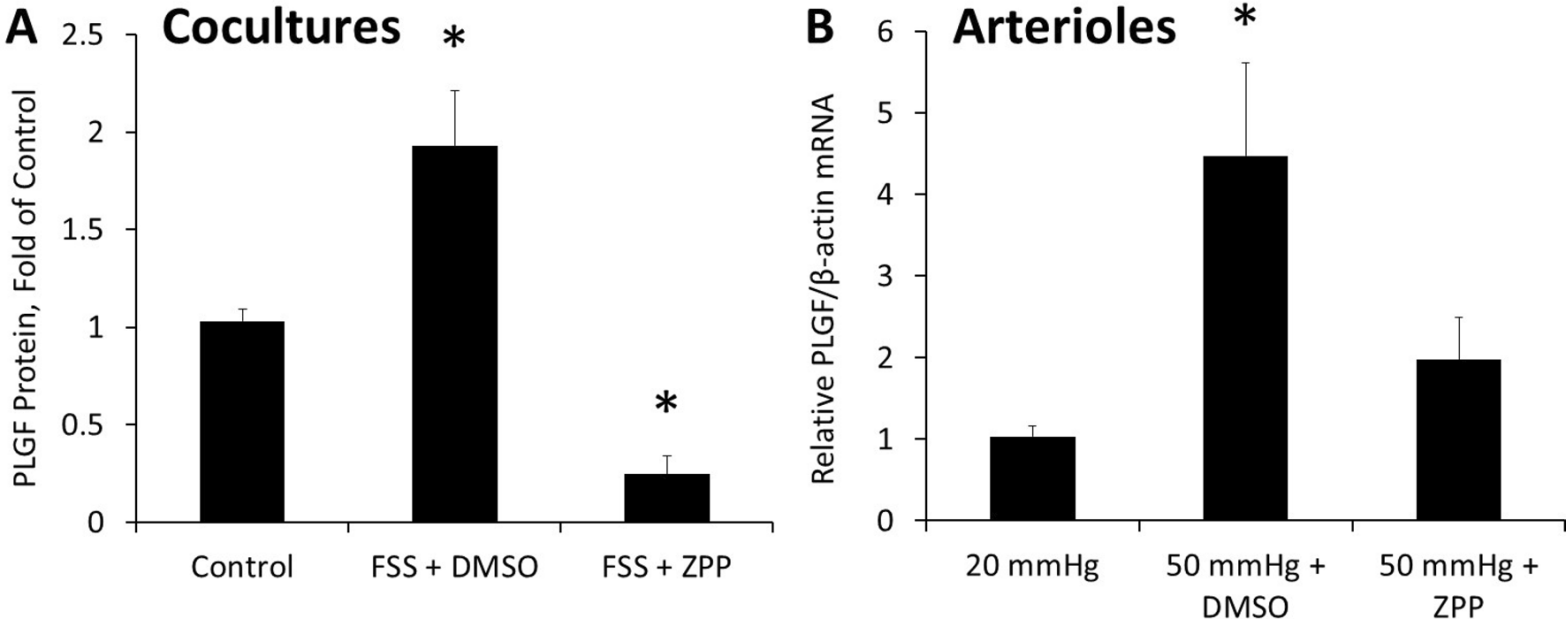
HO-1 activity is necessary for FSS to increase PLGF expression. (**A**) In co-cultures, ZPP (30 µM) inhibited the increase in secreted PLGF observed following exposure to FSS (n = 5, p < 0.05). (**B**) In mesenteric arterioles, perfusion at a pressure gradient of 50 mm Hg for 2 h increased PLGF mRNA compared to perfusion at 20 mmHg. This increase was blunted by ZPP (30 µM) (n = 5, p < 0.05).

We previously reported that FSS increases PLGF expression both in our co-culture model and in perfused vessels^24^. To determine HO-1’s role in the FSS induced expression of PLGF, we inhibited HO-1 with zinc protoporphyrin IX (ZPP). ZPP (30 µM) blocked the effect of FSS on PLGF, and even decreased PLGF protein below static control levels (Fig. 2A). Similarly, in perfused vessels ZPP (30 µM) prevented the effect of increased flow on PLGF mRNA (Fig. 2B).

**Figure 3:**
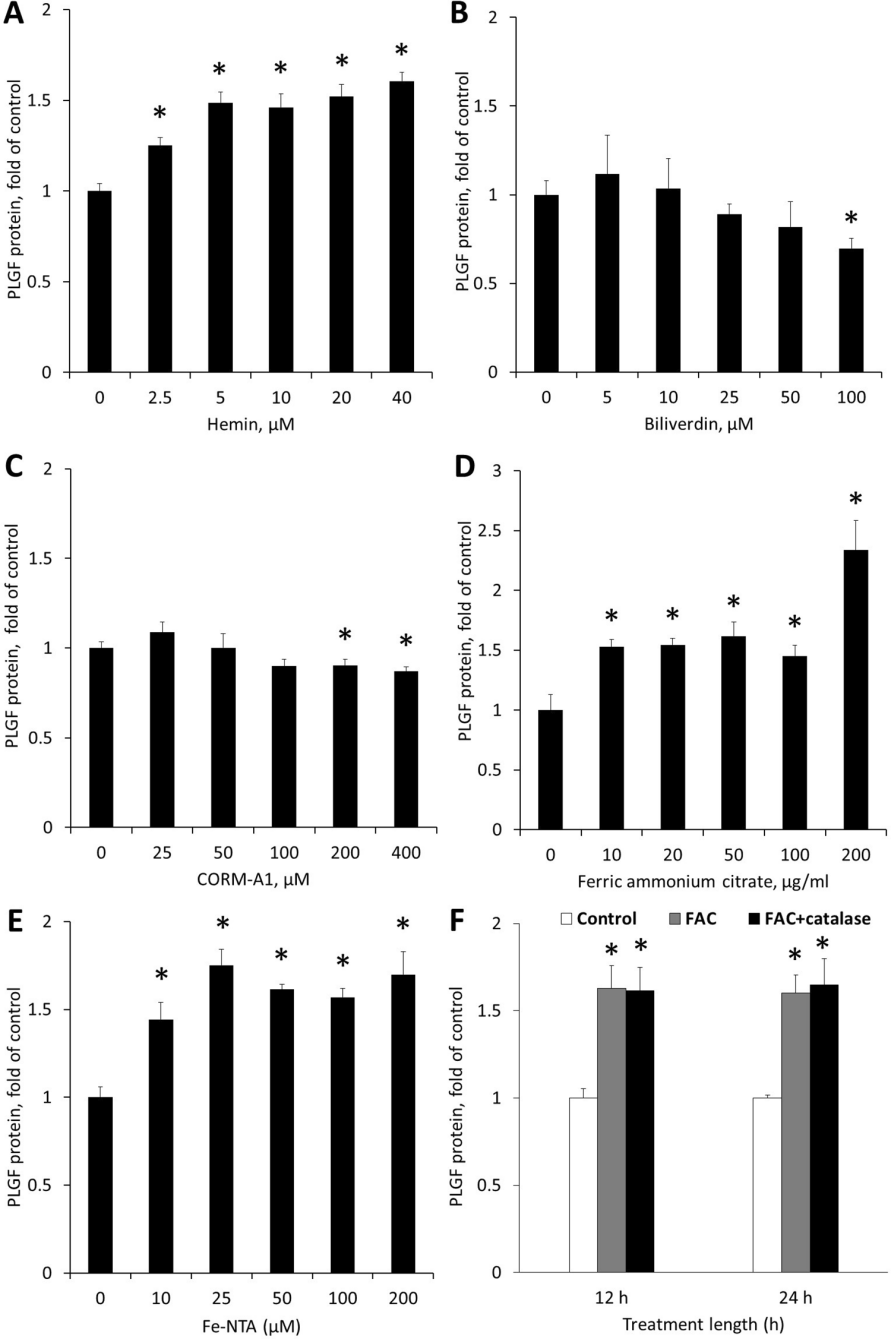
HO-1 activity increases secreted PLGF. Co-cultures were treated with the compounds shown for 24 h. (**A**) Hemin, an inducer of HO-1 activity, significantly increased secreted PLGF at all concentrations used (n = 5, p < 0.05). (**B**) Biliverdin, a product of HO-1, significantly decreased secreted PLGF at 100 µM (n = 5, p < 0.05). (**C**) CORM-A1, a carbon monoxide releasing molecule, slightly but significantly decreased secreted PLGF at 200 µM and 400 µM (n = 5, p < 0.05). (**D**) Ferric ammonium citrate (FAC) significantly increased secreted PLGF at all concentrations (n = 5, p < 0.05). (**E**) Iron nitrilotriacetic acid (iron-NTA) similarly increased PLGF levels. (**F**) Catalase treatment (500 U/mL) did not prevent FAC (100 μg/mL) from increasing PLGF (n = 4, p < 0.05).

In agreement with the above results, stimulating HO-1 activity with hemin chloride (2.5–40 µM) significantly increased PLGF protein levels in co-culture media after 24 h (Fig. 3A). HO-1 activity generates three products (biliverdin, carbon monoxide, and free iron). We next tested whether one or more of these molecules could upregulate PLGF expression in vascular cell co-cultures. Treatment with 100 µM biliverdin (which is also a negative feedback inhibitor of HO-1) significantly decreased PLGF protein (0.70 ± 0.06-fold of control) after 24 h (Fig. 3B). To determine whether CO modulates PLGF expression, we treated co-cultures with a CO-releasing molecule (CORM-A1, 0–400 µM) for 24 h (Fig. 3C). Similar to biliverdin, CORM-A1 significantly decreased secreted PLGF at the 200 µM (0.90 ± 0.03-fold of control) and 400 µM (0.86 ± 0.02 fold of control). Lastly, we tested the effect of iron on PLGF expression. Trivalent iron in the form of ferric ammonium citrate (FAC, 10–200 µg/mL) mimicked the effects of hemin chloride and significantly increased PLGF levels after 24 h (Fig. 3D). Iron nitrilotriacetic acid (iron-NTA, 10–200 µM) similarly increased PLGF levels (Fig. 3E). Catalase treatment (500 U/mL) had no effect on FAC-induced PLGF upregulation (Fig. 3F).

**Figure 4:**
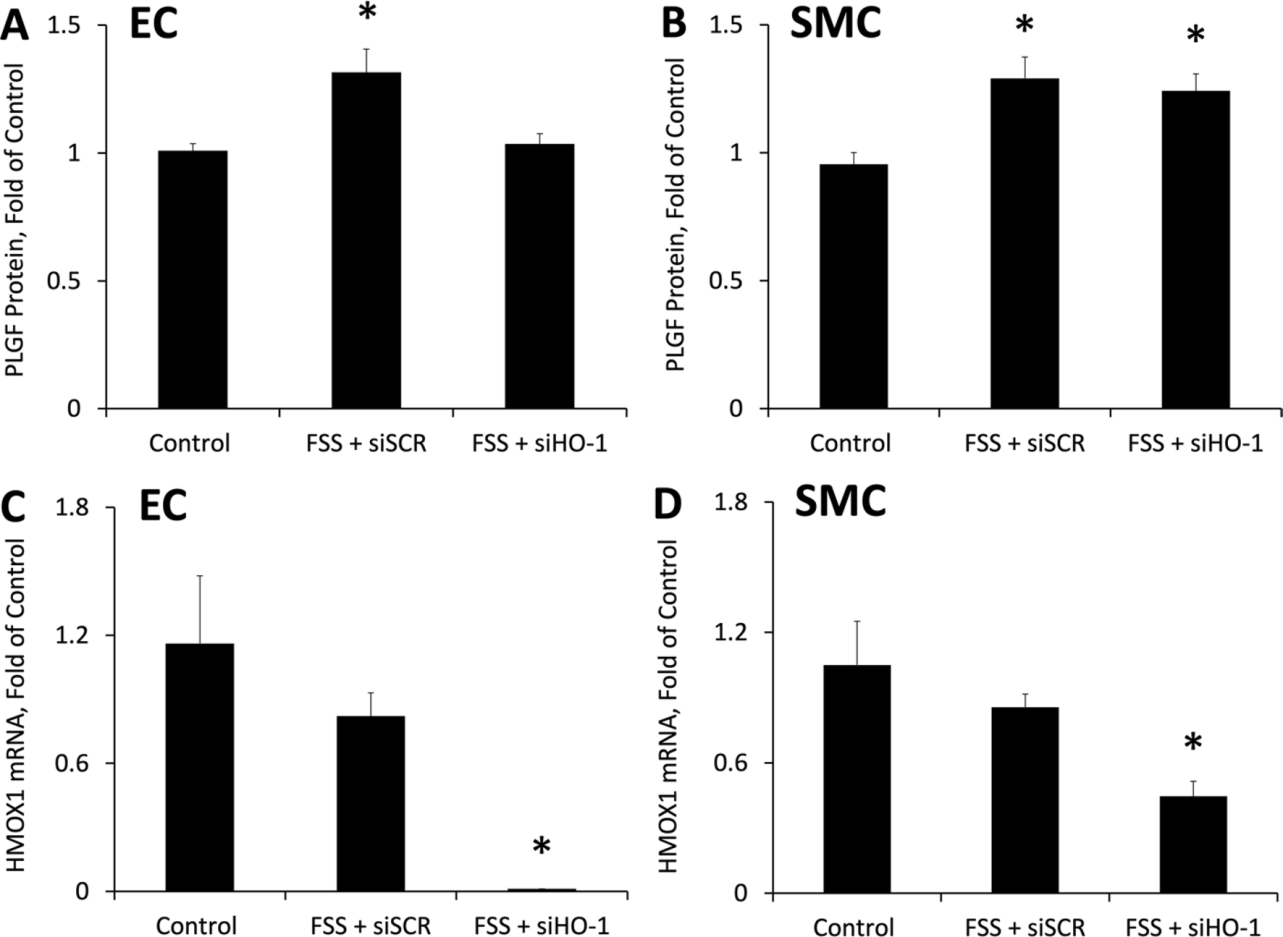
Endothelial HO-1 is necessary for FSS to increase PLGF expression. siRNA knockdown was performed on either the endothelial cells or the smooth muscle cells in the co-culture model. (**A**) Knockdown of HO-1 in endothelial cells blocked the effect of FSS to increase PLGF in co-cultures (n = 5, p < 0.05). (**B**) Knockdown of HO-1 in smooth muscle cells had no effect on FSS-induced upregulation of PLGF in co-cultures (n = 5, p < 0.05). (**C**) HO-1 knockdown in endothelial cells significantly decreased HO-1 mRNA in EC (n = 5, p < 0.05), but did not affect HO-1 mRNA in co-cultured SMC (not shown). (**D**) HO-1 knockdown in smooth muscle cells significantly decreased HO-1 mRNA in SMC (n = 5, p < 0.05), but did not affect HO-1 mRNA in co-cultured EC (not shown).

To determine the relative importance of endothelial cell vs smooth muscle cell HO-1 activity in mediating the effects of FSS on PLGF expression, HO-1 was separately knocked down in each cell type in the co-culture model using siRNA (Fig. 4). HO-1 knockdown in endothelial cells prevented the FSS-induced expression of PLGF (Fig. 4A). In contrast, knockdown of HO-1 in smooth muscle cells did not affect the FSS-mediated increase in PLGF (Fig. 4B). Knockdown in each cell type was confirmed by real time PCR (Fig. 4C,D). HO-1 knockdown in endothelial cells did not affect HO-1 mRNA in co-cultured smooth muscle cells, or vice versa (not shown).

## Discussion

In this study we demonstrated that FSS increases HO-1 expression both in an in vitro co-culture model of the cell wall and ex vivo in intact vessels. We further showed that the FSS mediated increase in PLGF expression which we previously reported is dependent on HO-1 activity, we also identified endothelial cell HO-1 activity as necessary for this response. FSS-independent activation of HO-1 (by hemin) was sufficient to induce PLGF expression. Furthermore, treatment of vascular cell co-cultures with the three products of HO-1 activity identified iron as the mediator of the effects of HO-1 on PLGF expression.

Blood flow recovery in the ischemic hindlimb of HO-1 knockout mice is significantly impaired^33^ and inhibition of HO-1 activity following hindlimb ischemia in mice results in poor flow recovery and diminished recruitment of circulating cells to the ischemic hindlimb^28^. Consistent with these observations, we found that inhibition of HO-1 activity attenuates the effects of shear stress on PLGF. PLGF stimulates collateral remodeling by recruitment of monocytes^6^ and other circulating cells to the vessel wall^34^. Therefore, our findings suggest a novel mechanism for the above-described effects of HO-1 inhibition on arteriogenesis in rodent models. Stimulation of HO-1 with cobalt protoporphyrin IX following myocardial infarction has been shown to result in improved outcome and greater neovascularization in rats^35^. Likewise, overexpression of HO-1 in both mice and rats results in improved re-establishment of blood flow following hindlimb ischemia^36,37^. Similarly, overexpression of PLGF has been shown to improve cardiac performance and vascularization following myocardial infarction in mice^38^.

We report that arteriogenic FSS increases HO-1 expression in endothelial cells and smooth muscle cells, consistent with previous findings that laminar FSS induces HO-1 in endothelial cells^39^. Increased flow induced HO-1 more robustly in intact vessels than did increased shear in our in vitro co-culture model. The co-culture model much more closely approximates the vessel wall than do monocultures of vascular cells^40^. However, there are limitations to the model, including the lack of exposure of the cells to cyclic tangential or circumferential stretch. Furthermore, the model lacks the complexity of the in vivo extracellular matrix, including the extensive glycocalyx of intact vessels (which is important in the mechanosensing machinery of the vessel wall)^41–44^. Despite these shortcomings, the co-culture model offers practical advantages with regard to cell type specificity for both genetic manipulation and assays, which are not easily achieved in whole tissue. Comparison of the results from the intact vessel model with those from the co-culture model suggests that the role of FSS in regulation of PLGF may be even more pronounced in vivo, highlighting the potential physiological significance of this pathway.

HO-1 catabolizes heme into three products: biliverdin, CO, and divalent iron. Biliverdin is subsequently metabolized into bilirubin by biliverdin reductase. Biliverdin exhibits strong antioxidant effects^45–47^ and has been demonstrated to inhibit ROS-induced angiogenesis in tumor cells^48^. Furthermore, biliverdin inhibits neointimal thickening following vascular injury in rats^49^. These beneficial effects have been attributed to biliverdin’s antioxidant properties. Consistent with its antioxidant action and its ability to inhibit HO-1, we found that biliverdin inhibited rather than enhanced PLGF expression in static co-cultures. We previously demonstrated that the effects of shear stress on PLGF expression are mediated by hydrogen peroxide produced by endothelial NADPH oxidase 4^24^; thus, antioxidants would not be expected to stimulate PLGF expression.

Several studies have demonstrated a cytoprotective role of HO-1^50,51^. The cytoprotective effects of HO-1 have been attributed to CO. Rats pretreated with the CO donor methylene chloride before myocardial infarction developed more intermediate and large collateral arteries in the infarct area^35^. Furthermore, CO has been reported to induce VEGF expression in endothelial cells^52^ and smooth muscle cells^53^. Despite these arteriogenic effects, the CO donor CORM-A1 did not induce PLGF expression in our co-culture model; indeed, it slightly but significantly reduced PLGF. CO has also been shown to have antioxidant properties^54–56^, which may act to reduce PLGF expression.

HO-1 activity results in the release of labile iron, which is cytotoxic in excess amounts. Increased labile iron leads to oxidative stress within the cell through Fenton reactions. We were able to demonstrate that the effects of iron on PLGF expression were not due to oxidative stress, as catalase treatment had no effect on the FAC-induced increase in PLGF. We conclude that iron is the HO-1 product that mediates upregulation of PLGF by FSS.

Increased iron levels in different pathological conditions including sickle cell disease (SCD)^57,58^ and hereditary hemochromatosis (HH)^59^ have been linked to significantly increased PLGF levels. More interestingly, PLGF levels in HH patients decreased after iron overload relief via phlebotomy^59^. Lower iron levels were associated with the risk of preeclampsia^60,61^, where low serum PLGF level is used as an important indicator for disease diagnosis. These studies provide an important aspect of the role iron plays in PLGF upregulation.

Iron metabolism is complex, and further alterations in iron homeostasis in our model system following FSS exposure remain to be determined. For example, as a protective response against pro-oxidant Fenton reactions, increased labile iron induces an increase in ferritin translation^62^ and iron sequestration. Labile iron also induces expression of an iron efflux pump colocalized with HO-1^63^. Ferroportin 1, an iron exporter, is also upregulated by labile iron generated by HO-1^64^. Indeed, HO-1 knockout in mice and HO-1 deficiency in humans is linked to anemia, due to accumulation of iron in tissues and decreased iron recycling^18,20,65^, whereas overexpression of HO-1 is associated with increased cellular iron efflux and decreased influx^66^. FAC has also been reported to increase ferritin synthesis^67^ and ferroportin expression^68,69^. Taken together, these data suggest that an increase in HO-1 expression/activity would be expected to result in a secondary decrease in labile or “chelatable” iron, which has implications for downstream signaling.

In conclusion, we demonstrate that the key arteriogenic factor PLGF is regulated by HO-1 in response to the physiological stimuli of FSS, which is considered to be an important signal for collateral development in the coronary circulation. This study builds on the increased interest in HO-1 and its metabolites in vascular remodeling by shedding light into a novel possible mechanism by which HO-1 exerts its arteriogenic effects.

## Methods

### Reagents

All reagents were purchased from Sigma-Aldrich unless otherwise specified.

### Perfused arterioles

All animal experimental procedures were approved by the Institutional Animal Care and Use Committee at Oklahoma State University (assurance number A3722-01, protocol number ACUP VM-15-13) and the reporting in this manuscript follows the recommendations in the ARRIVE guidelines. 6–8-week-old C57BL/6J male mice were purchased from Jackson Laboratories. Mice were deeply anesthetized with isoflurane delivered by a vaporizer and the heart was excised. The entire mesentery, with the superior mesenteric artery and vein, was dissected free and washed with 4 °C PBS. Second order mesenteric arterioles (≈ 120–180 μm) were isolated from the mesenteric tissue. The isolated arterioles were transferred to a vessel chamber at 4 °C (Living Systems Instrumentation). The chamber contained a pair of glass micropipettes and was filled with physiological saline solution (PSS) (142 mM NaCl, 4.7 mM KCl, 1.7 mM MgSO_4_, 0.5 mM EDTA, 2.79 mM CaCl_2_, 10 mM HEPES, 1.18 mM KH_2_PO_4_, pH 7.4). After cannulation of the proximal (upstream) end of the vessel, the intraluminal pressure was gradually raised (less than 20 mmHg) to clear the lumen of clotted blood. Once cleared, the distal (downstream) end of the vessel was also cannulated. Time from euthanasia to complete cannulation was under 60 min. The temperature of the bath was then raised to 37 °C and pressure was gradually raised to 60 mmHg (~ 10 mmHg/10 min). The pressure increase was achieved by gradually raising two reservoirs connected by silicone tubing to each cannula. Perfusion buffer consisted of 1% bovine serum albumin in PSS. Once equilibrated at 60 mmHg, the longitudinal pressure gradient was increased from zero to 20 mmHg (“control”) or 50 mmHg (“proarteriogenic”)^70^. This was achieved by lowering the distal reservoir and raising the proximal reservoir, allowing for the average intraluminal pressure to be maintained at 60 mmHg. The control flow rate was ~ 75 µL/min, and the “proarteriogenic” flow rate was ~ 170 µL/min. Vessels were then perfused for 2 h. Function of the vessel wall was determined at the end of perfusion by assessing the vasoconstrictive response to epinephrine and the vasodilator response to acetylcholine, as assessed by changes in vessel diameter measured by video micrometer.

### Cell culture

Human coronary artery endothelial cells (HCAEC) and human coronary artery smooth muscle cells (HCASMC) were purchased from Lonza. For HCASMC, donors included a 12 year old male, a 56 year old female, and a 30 year old male, while HCAEC donors were a 21 year old male and a 30 year old male. HCAEC were cultured in EBM-2 basal media supplemented with EGM-2MV SingleQuot factors (HCAEC complete media, Lonza). HCASMC were cultured in SMBM basal media supplemented with SmGM-2 SingleQuot factors (HCASMC complete media, Lonza). All cells were grown in a humidified incubator in 5% CO_2_ at 37 °C. Cells were used at passage five or six for all experiments. Serum reduced media for experiments to be performed in room air was prepared by diluting the appropriate complete media in low glucose DMEM (Hyclone, Fisher) at a ratio of 2:3 yielding a 2% serum media, and supplementing with 15 mmol/L HEPES.

### Co-culture model

**Figure 5:**
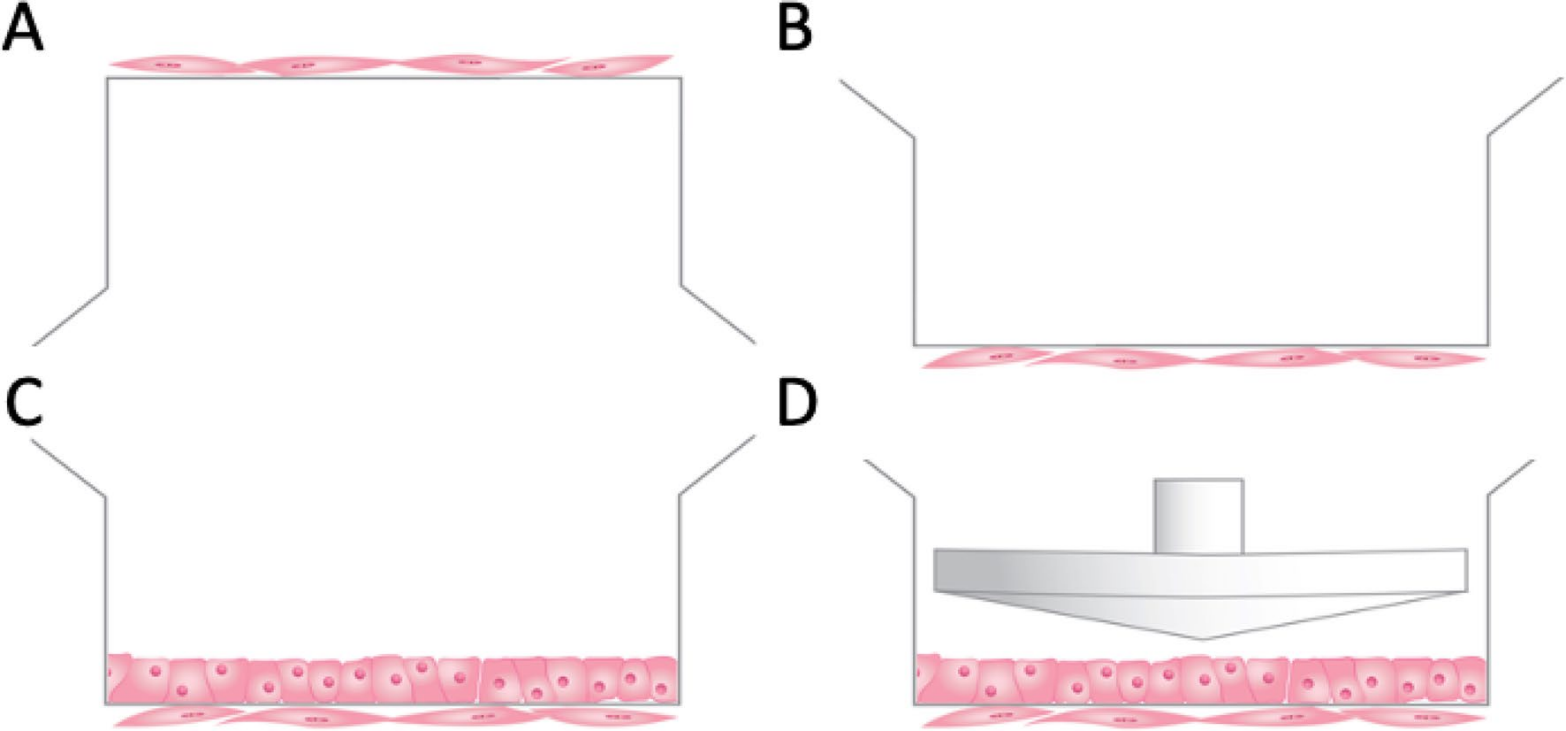
Illustration of in vitro co-culture model. (**A**) Transwell inserts were inverted and coated with gelation and HCASMC seeded onto the bottom of the insert. (**B**) After incubation overnight the iserts were reverted into 6 well plates overnight. (**C**) The top side of the insert was coat in gelatin and seeded with HCAEC and incubated overnight. (**D**) After a further overnight incubation in serum reduced media, cells were exposed to FSS using a microstepper driven cone.

Porous Transwell inserts (Corning Costar, 0.4 μm pore size) were used to model the vessel wall in vitro*,* as we previously described^24^. Inserts were inverted and the bottom surface was coated with 0.1% gelatin in DMEM, then were placed in a humidified incubator in 5% CO_2_ at 37 °C for 1 h. HCASMC (10^4^ cells/cm^2^) were then seeded onto the inverted insert, and inserts were returned to the incubator overnight (Fig. 5A). The following day, the inserts were placed into 6 well plates containing SmGM-2 media and incubated for an additional 24 h (Fig. 5B). The top surface of the insert was then coated with 0.1% gelatin in DMEM and incubated for 1 h. HCAEC (25,000/cm^2^) were then seeded on the top surface of the insert. EGM-2MV was added to the insert and the system was again incubated overnight (Fig. 5C). When possible, HCASMC and HCAEC donors were matched. Confluence of the co-cultures was confirmed by Hoffman modulation contrast microscopy (Olympus IX71). Lastly, the confluent co-cultures were incubated in serum reduced media for 24 h prior to experiments. For HCAEC mono-culture experiments, no HCASMC were seeded on the bottom of the insert, but cultures were otherwise processed as described above.

### Shear stress exposure

Only the HCAEC layer of the co-culture was directly exposed to FSS. FSS was applied using a cone and plate viscometer shearing system as we previously described^24^. Co-cultures were then exposed to a pulsatile FSS waveform that had a time-averaged FSS of 1.24 Pa (Fig. 5D). This waveform is based on previously published models of coronary collateral flow^71^. Shear experiments were performed on a laboratory benchtop in HEPES-buffered media with temperature maintained at 37 °C. Co-cultures were exposed to FSS for 2 h. Culture media and/or cell lysates were collected for analysis at various time points, from pre-shear up to 24 h post-shear.

### siRNA knockdown experiments

HCAEC were seeded into 6-well plates at a density of 210,000 cells/well. After 24 h, cells were transfected with either predesigned HO-1 siRNA (Silencer Select; s194530) or negative control siRNA (Silencer no.1 siRNA; scRNA), all purchased from Invitrogen. Prior to addition to cells, 5 nM of siRNA was precomplexed with lipofectamine RNAiMAX transfection reagent (Invitrogen) in Opti-MEM media (Gibco) for 20 min. Cells were exposed to transfection media (DMEM + 10% FBS containing precomplexed siRNA) for 6 h, after which cells were trypsinized and seeded onto the upper surface of inserts precoated with 0.1% gelatin. The lower surface of the inserts had been previously seeded with wild type HCASMC, as described above. Co-cultures were incubated overnight in reduced serum media as described above before exposure to shear stress. In a separate group of co-cultures, HCASMC were transfected similarly to HCAEC, after seeding onto the lower surface of inserts. At the end of the transfection period, HCASMC were washed and untreated HCAEC were then seeded onto the upper surface as above. Cell specificity and efficacy of target mRNA knockdown was determined by real time PCR.

### PLGF ELISA

Media samples were collected from treated cells and their corresponding controls, treated with protease inhibitor cocktail (1 mM PMSF, 1 mM Na_3_VO_4_, 1 µg/mL leupeptin, 1 mM benzamindine-HCl, 1 µg/mL aprotinin, 1 µg/mL pepstatin A) and stored at − 80 °C until further processing. Media samples were collected prior to treatment and 24 h after treatment (The time at which FSS had the greatest effect on PLGF protein) PLGF was measured using the DuoSet ELISA development kit (R&D Systems) according to manufacturer’s protocol. All samples were assayed in duplicate. Data were normalized to total protein concentration, as determined by BCA assay (Pierce). All assay plates were read on a Biotek Synergy HT plate reader.

### Real time PCR

After exposure to shear stress and media collection, cells were rinsed gently with PBS (HyClone) and trypsinized (TrypLE Express, Gibco). Collected cells were then resuspended in 1% β-mercaptoethanol in RLT lysis buffer (Qiagen) and frozen at − 80 °C for later processing. Cannulated vessels were placed in RLT lysis buffer immediately after perfusion and sonicated on ice using a Model D 100 Sonic Dismembrator (Fisher). Total RNA was isolated using RNeasy mini columns (Qiagen) following manufacturer’s directions. Total RNA quantity and quality were determined spectrophotometrically using a Take3 Micro-Volume Plate in a Synergy HT plate reader (Biotek). Reverse transcription was carried out using the QuantiTect reverse transcription kit (Qiagen) following manufacturer’s instructions. Real time PCR was performed on an ABI 7500 Fast instrument (Applied Biosystems) using PerfeCTa SYBR Green FastMix, Low ROX (Quanta Biosystems). Relative abundance of target mRNA was determined using the comparative Ct method and the following primer pairs: human PLGF forward 5′-CCTACGTGGAGCTGACGTTCT-3′; reverse 5′-TCCTTTCCGGCTTCA TCTTCT-3′, : human HO-1 forward 5′-CTGCGTTCCTGCTCAACATC-3′; reverse 5′-GGCAGAATCTTGCACTTTGTTG-3′, Mouse PLGF forward 5′-CTGCTGGGAACAACTCAACAGA-3′; reverse 5′-GCGACCCCACACTTCGTT-3′, Mouse HO-1 forward 5′-TCGTGCTCGAATGAACACTCTG-3′; reverse 5′-AGCTCCTCAAACAGCTCAATGT-3′. Gene expression was normalized to β-actin, as amplified with the following primers: human β-actin forward, 5′-TGCCGACAGGATGCAGAAG-3′; reverse, 5′-CTCAGGAGGAGCAATGATCTTGAT-3′; mouse β-actin forward, 5′-AGTTCGCCATGGATGACGAT-3′; reverse, 5′-TGCCGGAGCCGTTGTC-3′. Relative gene expression was quantified using the ΔΔCt method.

### Statistical analyses

All data are presented as mean ± SEM. Experiments were replicated 4–5 times. Data were analyzed by either two-way repeated measures ANOVA or one-way ANOVA, as appropriate. Both were followed by Tukey's range test. The level of significance was set at p < 0.05.
